# Validity of Wearable Sensors at the Shoulder Joint: Combining Wireless Electromyography Sensors and Inertial Measurement Units to Perform Physical Workplace Assessments

**DOI:** 10.3390/s19081885

**Published:** 2019-04-20

**Authors:** Isabelle Poitras, Mathieu Bielmann, Alexandre Campeau-Lecours, Catherine Mercier, Laurent J. Bouyer, Jean-Sébastien Roy

**Affiliations:** 1 Centre for Interdisciplinary Research in Rehabilitation and Social Integration and Laval University, Quebec City, QC G1M2S8, Canada; isabelle.poitras.2@ulaval.ca (I.P.); mathieu.bielmann.1@ulaval.ca (M.B.); Alexandre.Campeau-Lecours@gmc.ulaval.ca (A.C.-L.); Catherine.Mercier@rea.ulaval.ca (C.M.); Laurent.Bouyer@rea.ulaval.ca (L.J.B.); 2 Department of Rehabilitation, Laval University, Quebec City, QC G1V0A6, Canada; 3 Department of Mechanical Engineering, Laval University, Quebec City, QC G1V0A6, Canada

**Keywords:** range of motion, electromyography, shoulder, work-related disorder, level of physical demand

## Abstract

*Background*: Workplace adaptation is the preferred method of intervention to diminish risk factors associated with the development of work-related shoulder disorders. However, the majority of the workplace assessments performed are subjective (e.g., questionnaires). Quantitative assessments are required to support workplace adaptations. The aims of this study are to assess the concurrent validity of inertial measurement units (IMUs; MVN, Xsens) in comparison to a motion capture system (Vicon) during lifting tasks, and establish the discriminative validity of a wireless electromyography (EMG) system for the evaluation of muscle activity. *Methods*: Sixteen participants performed 12 simple tasks (shoulder flexion, abduction, scaption) and 16 complex lifting tasks (lifting crates of different weights at different heights). A Delsys Trigno EMG system was used to record anterior and middle deltoids’ EMG activity, while the Xsens and Vicon simultaneously recorded shoulder kinematics. *Results*: For IMUs, correlation coefficients were high (simple task: >0.968; complex task: >0.84) and RMSEs were low (simple task: <6.72°; complex task: <11.5°). For EMG, a significant effect of weight, height and a weight x height interaction (anterior: p < 0.001; middle: p < 0.03) were observed for RMS EMG activity. *Conclusions*: These results suggest that wireless EMG and IMUs are valid units that can be used to measure physical demand in workplace assessments.

## 1. Introduction

Work-related upper extremity disorders represent a major and increasingly prevalent health problem in industrialized countries [1], as they incur important economic costs, lead to loss of productivity, affect workers’ quality of life and can lead to premature retirement [2]. The shoulder is the joint most frequently affected by work-related upper extremity disorders, with a prevalence varying between 18 and 26% [3,4]. Physical work-related factors such as overhead work (more than 60° of arm elevation) and tasks requiring significant force (e.g., more than 10% of the maximal voluntary contraction, or lifting objects weighing more than 20 kg) appear to be important risk factors in the development of work-related shoulder disorders [5,6,7].

To prevent work-related shoulder disorders, most interventions use workplace adaptations or educational programs to decrease the physical demands on the shoulder joint. However, the lack of clinical objective assessments makes it difficult to determine the interventions’ efficiency to decrease repeated or sustained arm elevations or at lowering strength requirements. The majority of workplace assessments are based on self-administered questionnaires, interviews or clinical observations [3]. A few studies have used camera-based motion capture systems to assess simulated working tasks in the laboratory to complement the usual workplace assessments with more objective data [8,9]. However, camera-based systems lack portability and can hardly be used outside the laboratory [10]. 

Accelerometers have been used for several years to assess physical activity in rehabilitation research; however they have been shown to lack precision for kinematics estimation (e.g., lower accuracy for increasing segment acceleration) [11]. To overcome this limitation, gyroscopes and magnetometers are now commonly added to the wearable units (called inertial measurement units or IMUs). By combining the signals of each sensor through optimized data fusion algorithms, a reasonably accurate estimate of IMU orientation can be obtained. Still, a recent systematic review on the psychometric evidence of IMUs for the assessment of joint movement has reported highly variable results when using IMUs to evaluate the shoulder joint. The main reason behind these findings was the difficulty in analyzing movements in more than one plane [12]. 

Recent improvements in IMUs hardware/software/data processing could result in improved validity for the evaluation of movements at the shoulder joint. The improved systems could therefore be an alternative to quantifying human movements outside the laboratory, i.e., in more ecological settings, such as actual work environments. At the shoulder, IMUs have been validated in several contexts but mostly during simple arm movements (movements performed in only one plane of movement: sagittal, frontal or transverse) [10]. Unfortunately, such movements are not representative of real work demands. The shoulder is the most mobile joint of the human body, and everyday tasks require that it performs complex 3D movements [3,13]. Few studies have validated IMUs for the shoulder joint during complex tasks (movements performed in more than one plane of movement) and have shown that validity is highly variable [14,15,16,17], as previously mentioned. 

Other physical factors such as increases in muscle activity and muscle fatigue are also associated with a higher risk of work-related shoulder disorders [6]. During laboratory assessments, wireless electromyography (EMG) systems are often used to quantify muscle activity and fatigue, and these systems are sometimes also used in the clinic. During arm elevation, shoulder muscles are highly requested to maintain joint stability, and having an adequate muscle activation pattern plays a major role in the prevention of injuries [18]. To the best of our knowledge, only two studies have identified the effects of arm elevation and weight lifting on muscle activation during simulated working tasks [19,20]. However, the effect of shoulder range of motion was not accounted for, which can be an important contributing factor to shoulder pain of manual handling workers. Also, wireless EMG systems lack validation in work contexts, limiting their current application in clinical and work environments. 

Therefore, the aims of this study are (1) to evaluate the criterion validity of a commercial IMU system (MVN Awinda system, Xsens) by comparing it to a camera-based laboratory motion capture system (Vicon), during isolated shoulder movements and complex upper extremity lifting tasks; and (2) to evaluate the discriminative validity of a wireless EMG system (Delsys Trigno; EMG activity of the anterior and middle deltoid muscles) by looking at its discriminative capacities according to shoulder range of motion (ROM) and lifting weights. The hypotheses are that: (1) criterion validity will be characterized as good to excellent (0.8 ≥ r ≤ 1.0, error of measurement ≤ 15°) for both simple movements and complex tasks, but errors of measurement for arm elevation will be larger in complex tasks; and (2) that EMG activity will increase with arm elevation and heavy weight lifting (p < 0.05), and the increase will be larger for the anterior deltoid, as it is the main shoulder muscle agonist for lifting in the sagittal plane. Aggregating the IMU and EMG data will provide a good estimate of shoulder physical demands in simple and complex tasks simulating the real work environment.

## 2. Materials and Methods

### 2.1. Participants

Sixteen healthy participants (eight males, 12 right-handed (1 ambidextrous), 26.4 ± 4.1 years, 1.73 ± 0.09 m of height) were recruited (sample size required for an effect size of 0.8, with α = 0.05 and 1-β= 0.95). Inclusion criteria were: (1) to be between 18 and 65 of age, and (2) absence of self-reported neurological or musculoskeletal conditions (pain, mobility limitations) that could interfere with task execution. All participants gave written informed consent prior to experiment onset; this study was approved by the local ethics committee (CIUSSS-CN; project #217-539). 

### 2.2. Instrumentation and Data Collection

Shoulder movements were recorded simultaneously with nine IMUs positioned at standardized locations on the upper body (MVN, Xsens Technologies, Enschede, The Netherlands) and nine Vicon MX cameras (seven MT40-S and two MT10- S cameras, Vicon Motion Systems Limited, Oxford, UK), respectively at 60 and 100 Hz. The IMUs were placed on: the head, shoulder (2), sternum, upper arm (2), forearm (2) and pelvis. IMUs were fixed with hook and loop straps around arms and on Lycra suit for the trunk in accordance with the sensors configuration recommended by Xsens [21]. Rigid triads of retroreflective markers were placed on the C7 spinous process, as well as on the right and left upper arms. Single markers were temporarily placed bilaterally on specific anatomical landmarks (sternal notch, lateral epicondyle, medial epicondyle and glenohumeral junction) for calibration. To record muscle activity, wireless surface EMG sensors (Trigno Wireless EMG system, Delsys, Boston, MA, USA) were positioned bilaterally on the anterior and middle deltoid muscles according to Seniam recommendations [22].

### 2.3. Study Design and Experimental Procedure

All participants took part in one testing session. Before the experiment began, the experimenter explained the protocol, answered questions and obtained written consent from each participant. Then, all participants filled a sociodemographic questionnaire and the Edinburgh Handedness Inventory. Wireless EMG sensors were placed after skin preparation (skin was cleaned by rubbing it with alcohol for 5 seconds). A signal preview from each sensor was used to assess signal quality. Three maximal voluntary contractions (MVC) were performed for each muscle (anterior and middle deltoids) with one-minute breaks in between. Thereafter, Xsens sensors and Vicon markers were placed on the upper body. Anthropometric measures (height, shoulder width, arm span, hip height, hip width, knee height, ankle height, foot size and sole height) were gathered and filled in the MVN Studio software (MVN studio software, v. 4.4.0, Xsens Technologies). An anatomical pose (participant standing straight looking forward, arms along body side, palms facing forward) was performed to calibrate the Vicon system and an N-pose (participant standing straight looking forward, 90° shoulder abduction, palms facing the ground) was held to calibrate the Xsens system. Then, the protocol was divided in two parts: 1) isolated shoulder and trunk movements, and 2) complex lifting tasks.

During part one, IMU validity was first assessed during simple shoulder movements. Three isolated movements (flexion, abduction and scaption) were performed 5 times each at 3 different joint angles (60, 90 and 120° measured with an inclinometer). Then, three combined trunk movements (anterior flexion, lateral bending and rotation) were repeated 5 times each while the shoulder was maintained in a 90° flexion (12 different movements * 5 repetitions = 60 trials per participant).

A standardized handling assessment system, Valpar 19 (Valpar International Corporation, Tucson, AZ, United States), was used to assess the validity of IMU and surface EMG during complex tasks. This system comprises a shelf with three levels (high [H]: 1.74 m; medium [M]: 1.25 m; low [L]: 0.46 m from the ground) where crates of different weight representing specific physical work demand levels (sedentary [2.3kg], light [6.8kg], moderate [13.6kg] and moderate to heavy [22.7kg]) can be placed [23]. Participants were asked to lift a crate from a step located 1.8 m in front of the shelf, move it to a specific location on the shelf, take a 3-second break with arms along the body and return the crate back to the starting position. They were asked to take the lifting strategy of their choice and a stepladder was made available to them to bring the crates to the high shelf, when/if necessary. Each lifting (i.e., each given height and weight combination) was repeated twice. To ensure participant safety, only sedentary and light weights were used for the medium and higher shelves (2 weights × 2 trials = 4 trials/shelf); all weights were used for the lower shelf (4 weights × 2 trials = 8 trials). Height and weight were randomly assigned for a total of sixteen trials.

### 2.4. Data Processing

Kinematic data of the right arm were processed for both motion capture systems. The data collected from Vicon was processed in the Nexus software (Vicon Motion Systems Ltd., Oxford, UK). Each trial was manually inspected, markers were labelled and gaps of 15 or less samples were linearly interpolated. The data were then digitally low-pass filtered at 8 Hz (Butterworth double-pass filter). Before calculating joint angles, sensor positions were re-initialized in N-pose. Then, joint angles at the shoulder joint were calculated relative to the position of the trunk and arm markers and by applying the ZYZ Euler rotation sequence.

Each Xsens sensor orientation was extracted from MVN Studio BIOMECH (Xsens Technologies, Enschede, Netherlands) and exported in ASCII in the form of a quaternion. The data were then imported into MATLAB R2017a (The Math Works Inc., Natick, MA, USA) and the quaternions were converted into a rotation matrix. Joint angles were then calculated relative to the orientation of the trunk and upper arm sensors. The rotation sequence ZYZ was also used to calculate Euler angles. 

**Figure 1:**
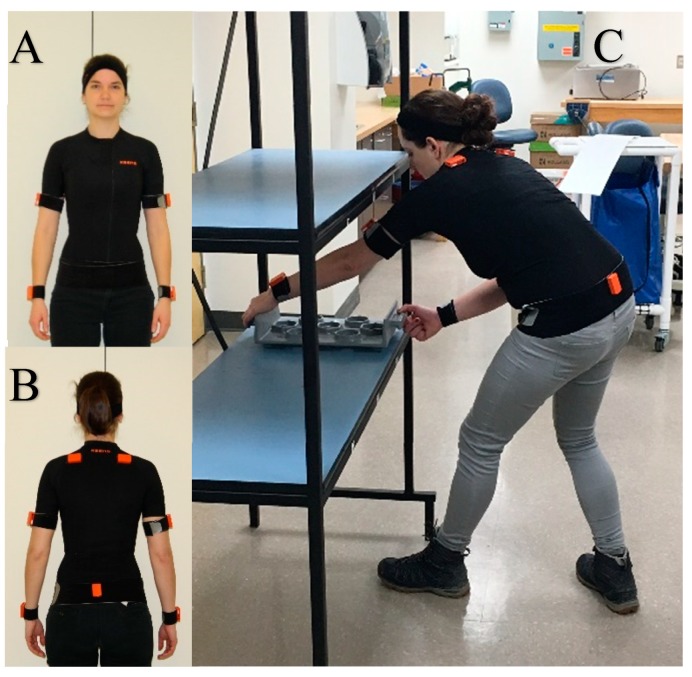
(**A**) Positioning example for sensors—front view, (**B**) Positioning example for sensors—back view, (**C**) Lifting trial example; for this specific trial, the left arm was analyzed as it was the arm which was farther from the body when lifting.

EMG signals were processed with a custom software written in MATLAB. They were digitally filtered with a fourth-order zero-lag Butterworth filter (band-pass 20–450 Hz), and a root-mean-square rectangular window of 20 ms was used for rectifying and smoothing the signal. Mean RMS value of MVC for each muscle was used to normalize EMGs. The output of accelerometers from Trigno and Xsens sensors was used to synchronize EMG and kinematic data by performing a cross-correlation. Then, a custom-written algorithm was used to identify the “lifting” and ”dropping” phases of the manual handling task. Analyses were only performed for the ”lifting” phase as both muscles perform concentric work during this phase of movement [24]. Results were calculated for the arm which was farther away from the body when lifting (identified manually for each trial, see Figure 1). The peak activation and the area under the burst were calculated for each weight and height.

### 2.5. Statistical Analysis

Descriptive analyses were performed on sociodemographic data (mean and standard deviation [SD]). Cross-correlation analyses (r) were performed on Vicon and Xsens data to establish the criterion validity of the Xsens for arm elevation. Correlation was considered as poor (less than 0.5), moderate (between 0.5 and 0.75), good (between 0.75 and 0.9) and excellent (greater than 0.90) [25,26]. Shoulder angles (each of the Euler angle separately) calculated from Vicon and Xsens for arm elevation were compared using root-mean-square error (RMSE) which represents the mean error between the angle calculated by the reference system and the IMU. For complex tasks, every trial was processed and analyzed separately; all results were then combined for statistical analysis. A one-way analysis of variance (ANOVA) was performed on kinematic data to test whether the RMSE differed according to the shelf height. A one-way ANOVA was also used to calculate the effect of lifting weight from the lower shelf on muscular activity (for peak value and root-mean-squared [RMS; area under the curve]). A two-way ANOVA was used to calculate the effect of shelf height (3 shelves), crate weight (2.3 and 6.8 kg) and crate weight x shelf height interaction effects on EMG activity (for peak value and RMS). 

## 3. Results

### 3.1. Kinematic Data

**Figure 2:**
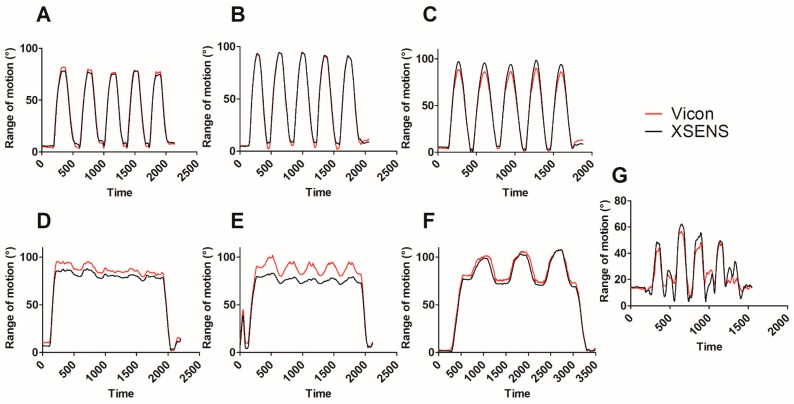
Kinematic pattern (range of motion) of a typical subject performing simple movements (**A**: 90° shoulder flexion, **B**: 90° shoulder abduction, **C**: 90° shoulder scaption, **D**: 90° shoulder flexion combined to trunk flexion, **E**: 90° shoulder flexion combined to lateral trunk bending, **F**: 90° shoulder flexion combined to trunk rotation) and a complex task (**G**: lifting and dropping of a crate on the medium shelf). Vicon kinematic pattern is traced in red and Xsens kinematic pattern is traced in black.

The correlation coefficients were excellent for all simple movements (range 0.917–0.999). Correlation coefficients for complex tasks were lower but still considered good (0.846 ± 0.103). Simple shoulder movements without combined trunk motion showed the lowest RMSE and average error of estimate (RMSE = 2.8–6.7°; error of estimate= 2.4–5.5°), while more variability was present for combined trunk movement, particularly for trunk rotation and lateral bending (Rotation [mean ± standard deviation (SD)]: RMSE = 12.8 ±7.6°; average error of estimate = 10.2 ± 6.1°; Lateral bending [mean ± SD]: RMSE = 11.6 ± 5.6°; average error of estimate = 9.7 ± 4.9°) and for the complex lifting tasks ([mean ± SD]: RMSE = 11.5 ± 2.4°; average error of estimate = 9.2 ± 2.0°). See Table 1 for detailed results and Figure 2 for the kinematic patterns of a typical subject.

### 3.2. EMG Activity

#### 3.2.1. Anterior Deltoid

When comparing the four weights for the lower shelf, anterior deltoid EMG activity was found to significantly increase with crate weight (RMS: p<0.0001, η^2^ = 0.51, peak: p < 0.0001, η^2^ = 0.46). Post-hoc analyses showed that the EMG activity increased gradually across weights lifted (RMS [mean (SD)]: L1 = 32.4 (10.9)%; L2 = 49.6 (35.7)%; L3 = 67.9 (37.0)%; L4 = 90.7 (58.0)%) and the difference was significant when comparing all pairs of weights (L1-L2: p = 0.003; L1-L3: p = 0.001; L1-L4: p = 0.0003; L2-L3: p = 0.021; L2-L4: p = 0.0001; L3-L4: p = 0.03). 

**Figure 3:**
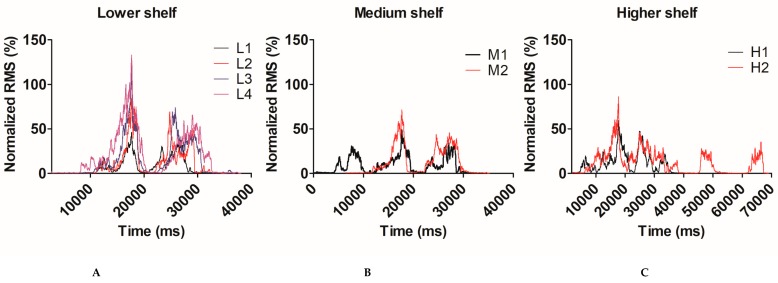
Anterior deltoid EMG activity (RMS) for a typical subject for different shelves (**A**: Lower shelf, **B**: Medium shelf, **C**: Higher shelf) and weights (Lower shelf: L1 = 2.3 kg, L2 = 6.8 kg, L3 = 13.6 kg, L4 = 22.7kg; Medium shelf: M1 = 2.3 kg, M2 = 6.8 kg; Higher shelf: H1 = 2.3 kg, H2 = 6.8 kg).

When comparing the two lightest weights (sedentary and light) across the three shelf heights, a significant effect of weight (RMS: p < 0.001, η^2^ = 0.89, peak: p = 0.001, η^2^ = 0.54) and height (RMS: p < 0.001, η^2^ = 0.48, peak: p = 0.003, η^2^ = 0.317) was found. A weight x height interaction was also observed (RMS: p=0.001, η^2^ = 0.36, peak: p=0.001, η^2^= 0.38). Post-hoc analyses revealed that the difference between the weights was significant only for the higher shelf (RMS [mean (SD)]: H1 = 64.90 (24.32%; H2 = 119.38 ± 44.66%, p < 0.001). Effects observed were larger for RMS data than peak EMG activity (for detailed results see Figure 3, Table 2; Table 3).

#### 3.2.2. Middle Deltoid

When comparing the four weights for the lower shelf, no significant difference was found for middle deltoid EMG activity (RMS: p = 0.159, η^2^ = 0.92, peak: p = 0.24, η^2^ = 0.09). When comparing results for the two lower weights on the three shelf heights, there was a significant effect found in the weight increase (RMS: p = 0.002, η^2^ = 0.523) and height increase (RMS: p = 0.028, η^2^ = 0.241), but only for the higher shelf (H1 vs H2: p < 0.001). Also, for the RMS, a weight, a height and a weight x height interaction effect were observed (weight: p = 0.002, η^2^ = 0.523; height: p = 0.028, η2 = 0.241; weight x height: p = 0.003, η^2^ = 0.352; height). For peak EMG activity, only a weight x height interaction was observed (p = 0.001, η^2^ = 0.388). Post-hoc analysis identified significant differences between L2-M2 (p: RMS = 0.002; peak = 0.013), M2-H2 (p: RMS = 0.004; peak = 0.015) and H1-H2 (p: RMS = 0.00045; peak = 0.001) for RMS and peak EMG. Also, a significant difference for L1-L4 peak EMG was observed (p = 0.027; for details, see Table 1; Table 2). 

## 4. Discussion

As we hypothesized, the results from this study confirm that the two commercial wireless systems (Xsens MVN and Trigno EMG) are valid tools to assess shoulder movements and muscle activity during simple arm elevations and complex lifting tasks. The comparison between Xsens and Vicon shows that IMUs are valid to assess shoulder elevation ROM during simple arm movements (r ≥ 0.917; RMSE ≤ 12.82°; average error of estimate ≤ 10.15°) and complex lifting tasks, regardless of the height at which the crates are placed on the shelves of Valpar 19 (r ≥ 0.839; RMSE ≤ 12.68°, average error of estimate ≤ 10.24°). As for EMG activity, the anterior deltoid should be considered as an interesting muscle to discriminate between the different levels of physical work demands (sedentary, light, moderate and high) when performing tasks that necessitate forceful contractions in the sagittal plane. The middle deltoid EMG activity varied depending on the weight and height at which the crates were placed, but was less discriminant than the anterior deltoid (significant results only for L2-H2, M2-H2 and H1-H2) for tasks performed in the sagittal plane. RMS and peak EMG activity were both discriminative. However, the discriminative potential of RMS is larger and it should therefore be preferred over peak activity to discriminate between the different physical work demands.

Assessing arm elevation is challenging since the shoulder is a highly mobile joint and necessitates 3D analysis. As mentioned above, IMUs could potentially be useful for workplace evaluations to quantify physical work demands over extended periods of time. However, no clear conclusion has previously been reached on IMU validity for arm elevation due to the heterogeneity of results and the small number of studies addressing their validity during complex tasks [10]. Our results confirm that IMUs are valid to assess arm elevation, although the correlations with the Vicon were higher for simple movements than for complex tasks. These results are compatible with previous studies in which the validity of maximal range in shoulder elevation (simple task) was evaluated [27,28,29,30]. However, two studies reported lower RMSE (ranging from 1.13 to 2.38°) than those reported herein with regards to arm elevation during simple movements [31,32]. These differences could be explained by several factors including differences in the biomechanical models used, sensor positioning and experimental protocols. Indeed, one study [31] reported results with the elbow constrained in the neutral position, thereby reducing errors in sensor misalignment due to soft tissue artifact. The second article [32] presented results during isolated wrist movements with small movements at shoulder joint. In comparison, our results are more representative of movement patterns seen in real life since the motion was not restricted. 

For complex tasks, two previous studies evaluated lifting tasks similar to those presented herein; however, their results were quite different [14,15]. One study [15] showed RMSE results lower than ours in regards to MVN Xsens. A post-processing step was however added to reduce the error due to the choice of the biomechanical model. The RMSE that they reported without removing biomechanical model variability was much larger than the RMSE reported in this study (19.7° vs. 2.9°, respectively). The other study [15] reported higher errors of measurement and more variability (ranging from 9.6 to 33.1°), but their results originate from using an older version of the XSens IMUs which could explain the discrepancy. 

It is well-known that performing physically demanding tasks at a shoulder level represents an important risk factor for developing work-related shoulder disorders [3]. Our results demonstrate that anterior deltoid muscle activity can be a good indicator of the physical demands on the shoulder since RMS and peak EMG activity can discriminate between different work demand levels (sedentary, light, moderate and heavy) [23]. These results correspond to those of a previous study from Silvetti et al, who observed an effect of weight and height on anterior deltoid activity [33]. Unlike this work, their study did not show a weight x height interaction, which could be explained by two main factors: 1) they placed the crates at varying distances from the shelf edge depending on the height at which it had to be placed (whereas we always used the same distance), and 2) the difference in weight between the two objects carried by the participants of Silvetti’s study was smaller (Silvetti’s study: 6/8 kg compared to 2.3/6.8 kg in this study). Furthermore, two other studies have shown results (height and weight effects) similar to ours regarding the anterior deltoid muscle, but they also found larger differences across weight lifted for the middle deltoid [19,20]. This difference could be explained by the fact that we only analyzed the lifting phase; they demonstrated higher co-activation during dropping than lifting. However, similar to this study, they have shown discriminative anterior deltoid potential. Therefore the results of the present study show that is possible to identify physical work demands of a specific task by analyzing muscle activity. 

### 4.1. Technical Issues to Be Considered Prior to Clinical Implantation

Other wearable sensors are also available to collect different types of bio-signals on workers (i.e., photosensors (light level), heart rate and skin conductance sensors (for stress and other physiological /psychological states), global positioning systems (GPS; mobility, environment). However, we chose to focus on the validation of IMU and EMG sensors as we consider them to have the highest potential for directly quantifying physical work demands at the upper extremities (e.g., number of arm elevations, time spent with the arm elevated, forceful work and muscle fatigue) [11]. Wearable technologies have greatly improved over the last few years. Progress has been made to improve software accuracy (e.g., development of better algorithms) and hardware stability (e.g., lesser sensitivity to magnetic disturbances [34]). Furthermore, real-time processing now allows to obtain more accurate 3D data with negligible processing delay. Systems have also significantly improved their portability given the development of smaller, lighter, wireless units. For all of these reasons, the two systems are potentially useful tools for clinicians. Still, certain technical concerns should be addressed before implementing them for workplace assessment. Indeed, the software used for data collection and the post-processing needed to obtain interpretable data are complex, time-consuming and not user-friendly (requiring technical competency in signal processing). The current technology therefore needs to be improved before clinical uptake. Also, and more specifically for IMUs, more flexibility in the number of sensors needed to collect meaningful and accurate data should be addressed as Xsens software requires at least seven IMUs. Nevertheless, the results for simple and complex movements are very promising for clinical use as the reported errors (RMSE ≤ 12.68°) are lower than the errors of inclinometers and goniometers currently used in clinic (95% limits of agreement ranged from 2° to 20°) [35]. Moreover, inclinometers and goniometers do not allow continuous monitoring during the task.

### 4.2. Study Limitations

The evaluation of shoulder kinematics is complex, and certain limitations need to be considered in our protocol. First, the calibration of both systems had to be performed at different body positions due to software requirements (N-pose for Xsens and anatomical calibration for Vicon) which can increase the error of measurement as validity is dependent on the calibration method [36]. We performed a post-processing re-initialization in N-pose to diminish the errors. Secondly, only arm elevation was analyzed. This choice was motivated by the clinical relevance of this movement, since the range of motion in shoulder elevation is usually targeted in workplace prevention/interventions. 

However previous studies suggest that the errors are usually higher for the other two rotations [10], and therefore the current results cannot be extended to other types of movement. For EMG activity recording, there is one main consideration regarding the protocol used. Considering the high prevalence of rotator cuff injury, it would have been interesting to evaluate rotator cuff muscles [37]. However, surface EMG recordings for rotator cuff muscles is not reliable since they are deep muscles [38]. Anterior and middle deltoids were the most appropriate superficial muscles to evaluate physical work demands as they showed greater amount of fatigue on EMG recordings in comparison to other superficial muscles in a variety of tasks [39,40,41]. Finally, this study is the first step in the validation process of these wearable sensors. Indeed, in the present research, the suitability of combining IMU and EMG sensors has been demonstrated during simulated working tasks. The next step will be to validate their use in the workplace, in the frame of actual work situations.

## 5. Conclusions

In conclusion, wireless EMG and IMU systems can be used to assess two important risk factors during simple and complex working tasks: 1) working with arms above the head and 2) using forceful contractions. IMUs reported lower errors of measurement compared to most tools currently used in the clinic (goniometer and inclinometer). Still, certain improvements need to be implemented for the systems to become more accessible and easier to use by clinicians. In addition, anterior deltoid muscle activity was shown to be a good indicator of physical demand. It is therefore a potentially useful clinical indicator to identify physically demanding tasks/jobs and to quantify muscle activity in the workplace. However, although these results are promising for socio-professional rehabilitation, studies performed in the workplace are now needed to support their suitability in actual work situations.

## Figures and Tables

**Table 1:** Correlation coefficient, root-mean-square error, average error of estimate and absolute error for simple and complex movements.

Movement/Task	Range of Motion (°)/Movement Combined	r (Mean [SD])	RMSE Arm Elevation (Mean [SD]) (°)	Average Error of Estimate (Mean [SD]) (°)
**Flexion**	60	0.968 [0.066]	5.17 [2.81]	4.38 [2.26]
	90	0.998 [0.002]	4.67 [2.95]	3.86 [2.33]
	120	0.997 [0.003]	6.21 [3.90]	4.97 [3.01]
**Abduction**	60	0.998 [0.001]	2.77 [1.28]	2.37 [1.13]
	90	0.999 [0.0003]	3.75 [2.86]	2.97 [2.18]
	120	0.999 [0.0004]	4.92 [3.02]	3.95 [2.35]
**Scaption**	60	0.997 [0.002]	3.95 [2.89]	3.17 [2.16]
	90	0.998 [0.001]	5.16 [4.12]	4.18 [3.45]
	120	0.999 [0.001]	6.72 [4.20]	5.46 [3.53]
**Trunk movements**	Anterior flexion	0.974 [0.057]	7.05 [3.81]	6.08 [3.53]
	Lateral bending	0.970 [0.041]	11.63 [5.56]	9.72 [4.85]
	Rotation	0.917 [0.099]	12.82 [7.61]	10.15 [6.12]
**Complex tasks**	Mean	0.846 [0.103]	11.48 [2.42]	9.18 [2.02]
	Lower shelf	0.851 [0.111]	9.62 [3.79]	7.77 [2.99]
	Medium shelf	0.840 [0.087]	11.33 [4.04]	9.03 [3.01]
	Higher shelf	0.870 [0.057]	12.68 [2.96]	10.24 [2.35]
	p-value	0.621	0.067	0.054

Legend: r: correlation coefficient; SD: standard deviation; RMSE: root-mean-square error.

**Table 2:** Mean and SD of normalized EMG activity (area under the curve and peak EMG activity) for anterior and middle deltoids.

			L1	L2	L3	L4	M1	M2	H1	H2
**Anterior deltoid**	RMS (% of MVC)	Mean	32.41	49.60	67.86	90.70	45.97	65.88	64.90	119.38
		SD	10.85	35.67	37.00	58.02	40.89	40.31	24.32	44.66
	Peak EMG activity (% of MVC)	Mean	37.93	65.45	75.71	87.35	39.91	54.02	56.66	87.28
		SD	13.05	44.40	42.11	50.49	30.79	35.68	17.90	34.74
**Middle deltoid**	RMS (% of MVC)	Mean	35.13	43.16	34.04	46.63	32.92	55.52	50.83	100.87
		SD	28.39	32.20	40.91	44.91	32.51	46.14	32.17	62.23
	Peak EMG activity (% of MVC)	Mean	38.84	50.08	47.55	53.86	27.63	43.64	41.39	68.50
		SD	29.15	42.58	38.14	36.28	23.21	33.61	26.19	40.30

Legend: SD: standard deviation; MVC: Maximum voluntary contraction; L1: lower shelf, weight 2.3kg; L2: lower shelf, weight 6.8 kg; L3: lower shelf, weight 13.6 kg; L4: lower shelf, weight 22.7kg; M1: medium shelf, weight 2.3 kg; M2: medium shelf, weight 6.8 kg; H1: higher shelf, weight 2.3 kg; H2: higher shelf, weight 6.8 kg.

**Table 3:** Results for anterior and middle deltoids’ EMG activity (p-value and effect size for area under the curve and peak EMG activity).

			One-Way ANOVA p-value	One-Way ANOVA η^2^	Two-Way ANOVA p-value	Two-Way ANOVA η^2^	Post-Hoc Analysis													
							L1 vs L2	L1 vs L3	L1 vs L4	L1 vs M1	L1 vs H1	L2 vs M2	L2 vs H2	L2 vs L3	L2 vs L4	L3 vs L4	M1 vs M2	M1 vs H1	M2 vs H2	H1 vs H2
**Anterior deltoid**	RMS (p-value)	Weight effect	<0.001	0.505	<0.001	0.889														
		Height effect	-	-	<0.001	0.480														
		Weight x Height effect	-	-	0.001	0.361	0.003	0.001	<0.001	0.042	0.197	0.928	<0.001	0.021	<0.001	0.03	0.124	0.678	<0.001	<0.001
	Peak EMG activity (p-value)	Weight effect	<0.001	0.460	0.001	0.540														
		Height effect	-	-	0.003	0.317														
		Weight x Height effect	-	-	0.001	0.383	0.077	0.001	<0.001	0.012	0.793	0.764	0.002	0.115	0.001	0.102	0.361	0.011	<0.001	<0.001
**Middle deltoid**	RMS (p-value)	Weight effect	0.159	0.917	0.002	0.523														
		Height effect	-	-	0.028	0.241														
		Weight x Height	-	-	0.003	0.352	0.143	0.791	0.626	0.302	0.834	0.723	0.002	0.754	0.810	0.576	0.378	0.325	0.004	<0.001
	Peak EMG activity (p-value)	Weight effect	0.244	0.093	0.121	0.163														
		Height effect	-	-	0.539	0.037														
		Weight x Height	-	-	0.001	0.388	0.647	0.274	0.027	0.206	0.194	0.788	0.013	0.783	0.562	0.383	0.408	0.054	0.015	0.001

Legend: RMS: root-mean-squared; η^2^: partial eta squared (effect size); L1: lower shelf, weight 2.3 kg; L2: lower shelf, weight 6.8 kg; L3: lower shelf, weight 13.6 kg; L4: lower shelf, weight 22.7 kg; M1: medium shelf, weight 2.3 kg; M2: medium shelf, weight 6.8 kg; H1: higher shelf, weight 2.3 kg; H2: higher shelf, weight 6.8 kg.
